# Poly(ADP-ribose) polymerase regulates transient receptor potential channel M2-dependent calpain activation in *rd1* mouse retinal degeneration

**DOI:** 10.4103/NRR.NRR-D-24-01102

**Published:** 2026-02-05

**Authors:** Jie Yan, Lei Kong, Zhijian Zhao, Qianlu Yang, Lan Wang, Qianxi Yang, Christian Harteneck, Kangwei Jiao, Zhulin Hu, François Paquet-Durand

**Affiliations:** 1 Yunnan Eye Institute & Key Laboratory of Yunnan Province, Yunnan Eye Disease Clinical Medical Center, Affiliated Hospital of Yunnan University, Yunnan University, Kunming, Yunnan Province, China; 2 Cell Death Mechanism Group, Institute for Ophthalmic Research, University of Tübingen, Tübingen, Germany; 3 High-resolution Functional Imaging and Test Group, Institute for Ophthalmic Research, University of Tübingen, Tübingen, Germany; 4 The Third Affiliated Hospital of Kunming Medical University &Yunnan Cancer Hospital, Kunming, Yunnan Province, China; 5 Department of Pharmacology and Experimental Therapy, Institute of Experimental and Clinical Pharmacology and Toxicology, University of Tübingen, Tübingen, Germany

**Keywords:** Ca^2+^, calcium channels, cyclic-guanosine-monophosphate (cGMP), PARthanatos, poly(ADP-ribose) polymerase-2 (PARP-2), retinitis pigmentosa

## Abstract

Inherited retinal degeneration refers to untreatable blinding diseases characterized by progressive photoreceptor loss. Photoreceptor degeneration is often associated with an excessive activation of poly(ADP-ribose) polymerase and Ca^2+^-dependent calpain-type proteases. To explore the interplay between poly(ADP-ribose) polymerase and calpain activity, we employed organotypic retinal explant cultures derived from wild-type mice and from the *rd1* mouse model for inherited retinal degeneration. Retinae were treated with the poly(ADP-ribose) polymerase inhibitors INO1001 or Olaparib, the poly(ADP-ribose) glycohydrolase inhibitor JA2131, or the transient receptor potential channel M2 blocker 8-Br-ADPR. Readouts included the terminal deoxynucleotidyl transferase dUTP nick end labeling assay to detect cell death, *in situ* activity assays for histone-deacetylases, poly(ADP-ribose) polymerase, and calpain, as well as immunostaining for activated calpain-2, and poly(ADP-ribose). Poly(ADP-ribose) polymerase, poly(ADP-ribose) glycohydrolase, and transient receptor potential channel M2 inhibition reduced calpain activity and calpain-2 activation. Poly(ADP-ribose) polymerase activity was decreased by poly(ADP-ribose) polymerase and transient receptor potential channel M2 inhibitors but not by poly(ADP-ribose) glycohydrolase inhibition. Remarkably, the poly(ADP-ribose) polymerase inhibitor INO1001 increased histone-deacetylase activity unlike any of the other compounds. When combined with the poly(ADP-ribose) glycohydrolase inhibitor JA2131, INO1001 reduced photoreceptor cell death in a synergistic fashion, although such synergy was not observed for calpain or poly(ADP-ribose) polymerase activity. Moreover, synergistic photoreceptor preservation was not observed when JA2131 was combined with the poly(ADP-ribose) polymerase inhibitor Olaparib. Overall, these results indicate that in *rd1* photoreceptors, poly(ADP-ribose) polymerase controls calpain activity via poly(ADP-ribose) glycohydrolase and transient receptor potential channel M2-induced Ca^2+^ influx. We also characterized INO1001 as potentially more beneficial for inherited retinal degeneration treatment than Olaparib. Our study details the complexity of poly(ADP-ribose) polymerase-signaling in photoreceptors and identifies poly(ADP-ribose) glycohydrolase and transient receptor potential channel M2 as new targets for inherited retinal degeneration therapy development.

## Graphical abstract

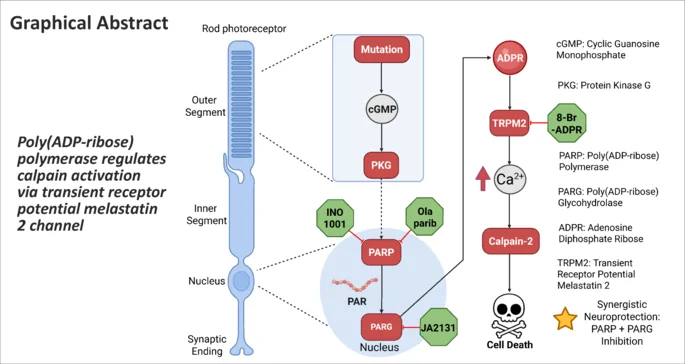

## Introduction

Inherited retinal degeneration (IRD) represents a diverse group of progressive, neurodegenerative diseases that often lead to blindness. IRD is characterized by progressive photoreceptor cell death (Botto et al., 2022) and is usually untreatable due to its genetic heterogeneity and lack of effective therapies. Within the IRD group, the most common disease is retinitis pigmentosa (RP) affecting approximately 1.5 million people all over the world (Cross et al., 2022). Early stage patients experience a loss of night vision due to the primary degeneration of rod photoreceptors. At late stages, the patient’s field of vision narrows until only central vision remains (i.e., “tunnel vision”). Eventually, the secondary degeneration of cone photoreceptors results in total blindness (Scalabrino et al., 2022). The second messenger cyclic-guanosine-monophosphate (cGMP) has been found to play a central role in the pathobiology of many genetically distinct types of IRD. In photoreceptor cells, excessive cGMP-signaling may be directly or indirectly related to the activity of poly(ADP-ribose) polymerase (PARP), Ca^2+^-dependent, calpain-type proteases and histone deacetylase (HDAC) (Yan et al., 2021).

The *rd1* mouse (retinal degeneration 1) is a laboratory mouse strain commonly used in research on retinal degeneration (Zhang et al., 2024). The *rd1* mouse carries a mutation affecting the expression of the beta subunit of phosphodiesterase 6 (PDE6). Since in rod photoreceptors, PDE6 beta is responsible for the hydrolysis of cGMP, the *rd1* mutation leads to an accumulation of cGMP and rapid rod loss (Cote, 2021). In photoreceptors, cGMP is known to act upon two prototypic targets: 1) the cyclic-nucleotide-gated (CNG) channel, which allows for Ca^2+^-influx and is the main effector of the phototransduction cascade, and 2) protein kinase G, which phosphorylates a large number of target proteins (Li et al., 2023). Probably further downstream from these two cGMP targets, cGMP accumulation in *rd1* mouse rods is associated with a prominent activation of PARP, calpain, and HDAC (Yan et al., 2022).

PARP is an enzyme family involved in DNA repair processes. When detecting DNA damage, PARP is activated and binds to the damaged sites, where it facilitates the recruitment of other DNA repair proteins to the site of damage (Lei et al., 2025). PARP uses nicotinamide adenine dinucleotide (NAD^+^) as a substrate to synthesize and attach poly(ADP-ribose) (PAR) chains onto itself and other target proteins. PAR polymers help to recruit and activate other DNA repair factors, creating a scaffold for efficient repair processes (Lei et al., 2025). Covalently attached PAR can be hydrolyzed by poly(ADP-ribose) glycohydrolase (PARG) to free PAR polymers or to mono adenosine diphosphate ribose (ADP-ribose [ADPR]) (Rouleau-Turcotte and Pascal, 2023). Paradoxically, excessive activation of PARP may also drive a specific form of cell death, termed PARthanatos, a phenomenon that has been linked to excessive NAD^+^ consumption or PAR generation (Zhang et al., 2025).

ADPR resulting from PARP activity is the main activator for transient receptor potential channel M2 (TRPM2), i.e., the transient receptor potential channel M2 (Maliougina and El Hiani, 2023). TRPM2 belongs to the transient receptor potential (TRP) superfamily of ion channels (Kashio, 2025). It is a Ca^2+^-permeable channel that is primarily expressed in immune cells, neurons, and various other tissues. Activation of the TRPM2 channel leads to an influx of Ca^2+^ ions into the cell, which, in turn, may trigger downstream signaling cascades and cellular responses (Huang et al., 2024; Shitaw et al., 2025). For instance, TRPM2-mediated Ca^2+^-influx can lead to the activation of Ca^2+^-dependent, calpain-type proteases that play important roles in various cellular processes such as cell signaling, cytoskeletal remodelling, cell migration, and programmed cell death (Ko et al., 2019). When Ca^2+^ binds to the Ca^2+^-binding domain within calpain enzymes, it triggers their activation and enables them to selectively cleave target proteins at specific sites (Metwally et al., 2023).

HDAC regulates gene expression by removing acetyl groups from histones, impacting chromatin structure and gene activity (Curcio et al., 2024). HDACs are categorized into two main groups based on their cofactor dependency: 1) Zinc-dependent HDACs: These include Class I, II, and IV HDACs. Class I HDACs (HDAC 1, 2, 3, 8) are primarily found in the nucleus and resemble yeast RPD3 deacetylase. Class II HDACs are further divided into IIa (HDAC 4, 5, 7, 9) and IIb (HDAC 6, 10), which shuttle between the nucleus and cytoplasm. Class IV comprises only HDAC 11, which shares features with both Class I and II. 2) NAD^+^-dependent HDACs: known as Class III or Sirtuins, these HDACs require NAD^+^ to function and differ significantly in mechanism and function from the zinc-dependent classes (Curcio et al., 2024). Over-activation of HDAC may trigger photoreceptor cell death (Dong et al., 2023a). However, neuroprotection exhibited by Sirtuins has sparked debate about the role of HDACs in RP (Yan et al., 2024).

Our previous research suggested that PARP and calpain take part in the same cell death pathway triggered by excessive cGMP-signaling (Yan et al., 2021). PARP appears to control calpain activity while calpain does not regulate PARP activation (Yan et al., 2022). Nevertheless, how exactly PARP regulates calpain activity is still unknown.

Here, we hypothesized that PARP may control calpain activation via ADPR-induced TRPM2 activation. To investigate this possibility, we used specific inhibitors for PARP and PARG, as well as an inhibitory ADPR analogue in organotypic *rd1* retinal explant cultures. Through these interventions, we show that (1) PARP, PARG, and ADPR can promote photoreceptor cell death; (2) PARP regulates calpain activity through ADPR-dependent activation of TRPM2; and that (3) the PARP inhibitor INO1001 displays superior therapeutic efficacy compared to the FDA-approved PARP inhibitor Olaparib.

## Methods

### Animals

For retinal explant cultures, C3H/HeA *Pde6b*^rd1/rd1^ animals (*rd1*), their congenic wild-type C3H/HeA *Pde6b*^+/+^ counterparts wild-type (wt) (Sanyal and Bal, 1973), and B6.129SvJ;C3H/HeA-*CNGB1*^tm^ double-mutant mice (rd1*Cngb1^−/−^) were used (Paquet-Durand et al., 2011). The rd1*Cngb1^−/−^ double mutants were generated by an intercross of *rd1* and *Cngb1*^−/−^ and have been maintained by repeated backcrossing over 10 generations to make a congenic inbred strain, homozygous for both gene mutations. The experimental animals were housed in the institutional, specified pathogen free (SPF) facility, with temperature between 21.5°C and 22.5°C, and relative humidity between 45% and 55%, under standard white cyclic lighting (12 hours/12 hours). The animals had free access to food and water and were used irrespective of sex. All animal procedures were conducted in strict accordance with both German and Chinese ethical standards. The protocols compliant with §4 of the German Animal Protection Act were reviewed and approved by the Tübingen University Committee on Animal Protection (Einrichtung für Tierschutz, Tierärztlicher Dienst und Labortierkunde; Registration numbers: AK02/19M, approved April 3, 2019; AK01/20M, approved February 17, 2020). Furthermore, the study was also supervised and approved by the Laboratory Animal Welfare and Ethics Committee of Yunnan University, ensuring compliance with their institutional guidelines (approval No. YNU20241125, approved December 23, 2024).

### Organotypic retinal explant culture

To study the effects of various drugs on photoreceptor enzyme activities and cell death, *wt*, *rd1*, and rd1*Cngb1^−/−^ retinas were explanted at postnatal day 5 (P5). The retinal explants were maintained in culture until either P11 (*wt*, *rd1*) or P17 (*wt*, rd1*Cngb1^−/−^). The explants were cultured on a polycarbonate membrane (83.3930.040; 0.4 µm TC-inserts, SARSTEDT, Hildesheim, Germany) with complete R16 medium (Gibco, Paisley, UK; with supplements) (Belhadj et al., 2020). During the cultivation, the complete R16 medium was changed every 2 days with treatment. The two retinas obtained from a single animal were split across different experimental groups so as to maximize the number of independent observations acquired per animal. Cultures were treated with 0.1 µM INO1001 (IC50: **~**3 nM (Pires et al., 2025), Cat# A20680, AdooQ, Irvine, CA, USA), 5 µM JA2131 (IC50: 0.4 μM (Houl et al., 2019), Cat# HY-137924; MedChemExpress, Sollentuna, Sweden), 50 µM 8-Br-ADPR (IC50: 5 μM (Partida-Sanchez et al., 2007), Cat# B 051, BIOLOG, Bremen, Germany), and 1 µM Olaparib (IC50: 5 nM (Menear et al., 2008), Cat# T3015, TargetMol, Boston, MA, USA) for monotherapies and 0.1 µM INO1001 and 5 µM JA2131 or 1 µM Olaparib and 5 µM JA2131 for combined therapies (for compound details see **Additional Table 1**). All compounds were dissolved in dimethyl sulfoxide (DMSO) at a final medium concentration of no more than 0.1% DMSO. Cultures were ended at P11 (*rd1*), and P17 (rd1*Cngb1^–/–^) by either fixation with 4% paraformaldehyde (PFA) or without fixation and direct freezing in liquid N2. Explants were embedded in Tissue-Tek (Sakura Finetek Europe B.V., Alphen aan den Rijn, The Netherlands) and sectioned (14 µm) in a cryostat (CryoStar NX50 OVP, Thermo Fisher Scientific, Runcorn, UK).

**Additional Table 1:** Information of compounds used in *rd1* explant cultures

Name	Structure	Targets	IC_50_	Reference
INO1001	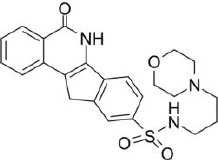	PARP-1 PARP-2	3-8 nM 220 nM	Komjáti et al., 2004; Pires et al., 2025
JA2131	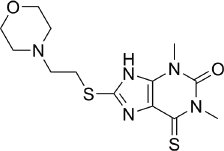	PARG	400 nM	Houl et al., 2019
8-Br-ADPR	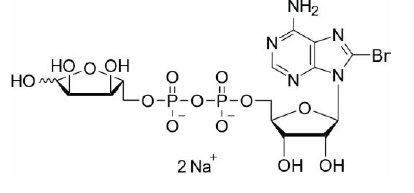	TRPM2	5 μM	Partida-Sanchez et al., 2007
Olaparib	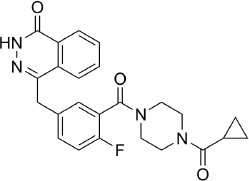	PARP-1 PARP-2 Tankyrase-1	5 nM 1 nM 1.5 μM	Senra et al., 2011; Yasukawa et al., 2016; Bian et al., 2018

8-Br-ADPR: TRPM2 blocker; INO1001: PARP inhibitor; JA2131: PARG inhibitor. PARG: poly(ADP-ribose) glycohydrolase; PARP: poly(ADP-ribose) polymerase; TRPM2: transient receptor potential channel M2.

### Terminal deoxynucleotidyl transferase dUTP nick end labeling assay

Terminal deoxynucleotidyl transferase dUTP nick end labeling (TUNEL) assay kit (Roche Diagnostics, Mannheim, Germany) was used to label dying cells. Histological sections from retinal explants were dried and stored at –20°C. The sections were rehydrated with phosphate-buffered saline (PBS; 0.1 M) and incubated with proteinase K (1.5 µg/µL) diluted in 50 mM TRIS-buffered saline (TBS; 1 µL enzyme in 1 mL TBS) for 15 minutes. This was followed by 3 times 5 minutes TBS washing and incubation with blocking solution (10% normal goat serum, 1% bovine serum albumin, and 1% fish gelatine in phosphate-buffered saline with 0.03% Tween-20). TUNEL staining solution was prepared using 21 parts of blocking solution, 18 parts of TUNEL labeling solution, and 1 part of TUNEL enzyme. After blocking, the sections were incubated with TUNEL staining solution overnight at 4°C. Finally, sections were washed 2 times with PBS, mounted using mounting medium with 4′,6-diamidino-2-phenylindole (DAPI; Cat# ab104139, Abcam, Cambridge, UK), and imaged by a Zeiss Imager Z.2 fluorescence microscope with ApoTome 2 (Carl Zeiss Microscopy, Oberkochen, Germany).

### Calpain activity assay

To visualize overall calpain activity *in situ* on unfixed tissue, retinal tissue sections were incubated and rehydrated for 15 minutes in calpain reaction buffer (5.96 g 4-(2-hydroxyethyl)-1-piperazineethanesulfonic acid, 4.85 g KCl, 0.47 g MgCl_2_, and 0.22 g CaCl_2_ in 100 mL double destilled water [ddH_2_O]; pH 7.2) with 2 mM dithiothreitol. Tissue sections were incubated for 3 hours at 37°C in calpain reaction buffer with 7-Amino-4-Chloromethylcoumarin, tert-butoxycarbonyl-L-Leucyl-L-Methionine amide (tBOC-Leu-Met-CMAC; 25 µM; Cat# A6520, Thermo Fisher Scientific). Then, sections were washed with PBS and incubated with ToPro (1:1000 in PBS, Thermo Fisher Scientific) for 15 minutes. Afterwards, tissue sections were washed twice in PBS (5 minutes) and mounted using Vectashield without DAPI (Vector Laboratories Inc., Burlingame, CA, USA) for immediate visualization by a Zeiss Imager Z.2 fluorescence microscope with ApoTome 2.

### Poly(ADP-ribose) polymerase activity and poly(ADP-ribose) staining

The PARP *in situ* activity assay is based on the incorporation of a fluorescent NAD^+^ analogue and allows resolving overall PARP enzyme activity on unfixed tissue sections (Belhadj et al., 2021). Such sections were incubated and rehydrated for 10 minutes in PBS. The reaction mixture (10 mM MgCl_2_, 1 mM dithiothreitol, and 50 μM 6-Fluo-10-NAD^+^ (Cat# N 023; BIOLOG) in 100 mM Tris buffer with 0.2% Triton X100, pH 8.0) was applied to the sections for 3 hours at 37°C. After three 5-minute washes in PBS, sections were mounted in Vectashield with DAPI (Vector) for subsequent microscopy by a Zeiss Imager Z.2 fluorescence microscope with ApoTome 2.

For the detection of PAR, we used an immunostaining enhanced by 3,3′-diaminobenzidine (DAB) staining. The procedure was initiated by quenching of endogenous peroxidase activity using 40% MeOH and 10% H_2_O_2_ in PBS with 0.3% Triton X-100 (PBST) in retinal tissue sections for 20 minutes. Sections were further incubated with 10% normal goat serum (NGS) in PBST for 30 minutes, followed by anti-PAR antibody (1:200, ALX-804-220-R100, Enzo Life Sciences, Farmingdale, NY, USA) incubation overnight at 4°C. Incubation with the biotinylated secondary antibody (1:150, Vector in 5% NGS in PBST) for 1 hour was followed by the Vector ABC Kit (Vector, solution A and solution B in PBS, 1:150 each) for 1 hour. DAB staining solution (0.05 mg/mL NH_4_Cl, 200 mg/mL glucose, 0.8 mg/mL nickel ammonium sulphate, 1 mg/mL DAB, and 0.1% glucose oxidase in phosphate buffer) was applied evenly, incubated for precisely 3 minutes, and immediately rinsed with phosphate buffer to stop the reaction. Sections were mounted in Aquatex (Merck, Darmstadt, Germany).

### Histone deacetylase activity assay

This assay allows detecting overall HDAC activity *in situ* on fixed tissue sections and is based on an adaptation of the FLUOR DE LYS®-Green System (Biomol, Hamburg, Germany). Retinal sections were exposed to 50 µM FLUOR DE LYS®-SIRT1 deacetylase substrate (BML-Kl177-0005; ENZO) with 2 mM NAD^+^ (BML-KI282-0500; ENZO) in assay buffer (50 mM Tris/HCl, 137 mM NaCl; 2.7 mM KCl; 1 mM MgCl_2_; pH 8.0) for 3 hours at 37°C. Sections were then washed in PBS and fixed in methanol at –20°C for 20 minutes. Slides were mounted with FLUOR DE LYS® developer II concentrate (BML-KI176-1250; ENZO) diluted 1:5 in assay buffer overnight for subsequent microscopy by a Zeiss Imager Z.2 fluorescence microscope with ApoTome 2.

### Immunohistochemistry for calpain-2 and transient receptor potential channel M2

Retinal tissue sections were rehydrated with PBS for 15 minutes and then incubated with a blocking solution (10% NGS, 1% BSA, and 0.3% PBST) for 1 hour at room temperature. The primary antibodies, rabbit-anti-calpain-2 (1:200, Cat# ab39165, Abcam), and rabbit-anti-TRPM2 (1:200, Cat# NB110-81601, Novus Biologicals, Centennial, CO, USA) were diluted in blocking solution and incubated overnight at 4°C rinsing with PBS for three times for 10 minutes each; this was followed by incubation with the secondary antibodies, goat-anti-rabbit AlexaFluor488 (1:400, Cat# A11034, Molecular Probes, Eugene, OR, USA), and goat-anti-rabbit AlexaFluor568 (1:300, Cat# A11036, Molecular Probes) for 1 hour at room temperature. The sections were rinsed with PBS three times for 10 minutes each and mounted with mounting medium with DAPI (Abcam). In some instances TRPM2 was co-labelled with cone photoreceptors using 1h (RT) incubation with fluorescently labeled peanut agglutinin (PNA; 1:500, Ca5# FL-1071, Vector Laboratories).

### Microscopy and image analysis in retinal cultures

Images of organotypic explant cultures were captured using a Zeiss Imager Z.2 fluorescence microscope, equipped with ApoTome 2, an Axiocam 506 mono camera, and HXP-120V fluorescent lamp (Carl Zeiss). Excitation (λExc.) and emission (λEm.) characteristics of filter sets used for different fluorophores were as follows (in nm): DAPI (λExc. = 369 nm, λEm. = 465 nm), AF488 (λExc. = 490 nm, λEm. = 525 nm), AF568 (λExc. = 578 nm, λEm. = 602 nm), and ToPro (λExc. = 642 nm, λEm. = 661 nm). The Zeiss Zen 2.3 blue edition software was used to capture images (tiled and z-stack, 20× magnification). Sections of 14 µm thickness were analyzed using 12–15 Apotome Z-planes. For quantification of positive cells in the outer nuclear layer (ONL), we proceeded as follows: The number of cells in six different rectangular ONL areas was counted manually based on the number of DAPI-stained nuclei, and used to calculate an average ONL cell size. This average ONL cell size was used to calculate the total number of cells in a given ONL area. The percentage of positive cells was then calculated by dividing the absolute number of cells positive for a given marker by the total number of ONL cells. The signal intensity on retinal sections was quantified by Zeiss Zen software version 3.0 (Carl Zeiss).

### Statistical analysis and software use

Two-way comparisons were analysed using Student’s *t*-test. Multiple comparisons were made using a one-way analysis of variance test with Tukey´s multiple comparison test. Calculations were performed with GraphPad Prism 8 (GraphPad Software, La Jolla, CA, USA); *P* < 0.05 was considered significant. Data in figure 5A was normalized by linear scaling according to the formula: *χ*_scaled_ = *χ* – *χ*_min_/*χ*_max_ – *χ*_min_, using SPSS Statistics 26 (IBM, Armonk, NY, USA). The figures were prepared using Photoshop 2022 and Illustrator 2022 (Adobe, San Jose, CA, USA). Spearman analyses were performed by R software (Version 4.0.1; R Foundation for Statistical Computing, Vienna, Austria; https://cran.r-project.org/src/base/R-4/).

## Results

### Transient receptor potential channel M2 is differentially expressed in wild-type and retinal degeneration 1 retina

The *rd1* photoreceptor degeneration begins around post-natal day (P) 9 followed by a significant rise of dying cells in the ONL (i.e., the photoreceptor layer) first seen at P11 (Wei et al., 2021). By P20 the *rd1* mouse retina had lost nearly all rod photoreceptors and a large fraction of cone photoreceptors. Our previous research had identified a strong up-regulation of PARP together with an accumulation of PAR in dying *rd1* photoreceptors (Paquet-Durand et al., 2007). Furthermore, we had shown that cGMP-dependent photoreceptor degeneration was strongly decreased after a knock-out of the PARG110 isoform (Sahaboglu et al., 2014). Since ADPR monomers generated by the combined activity of PARP and PARG can activate TRPM2, we hypothesized that TRPM2 could be involved in *rd1* degeneration.

**Figure 1:**
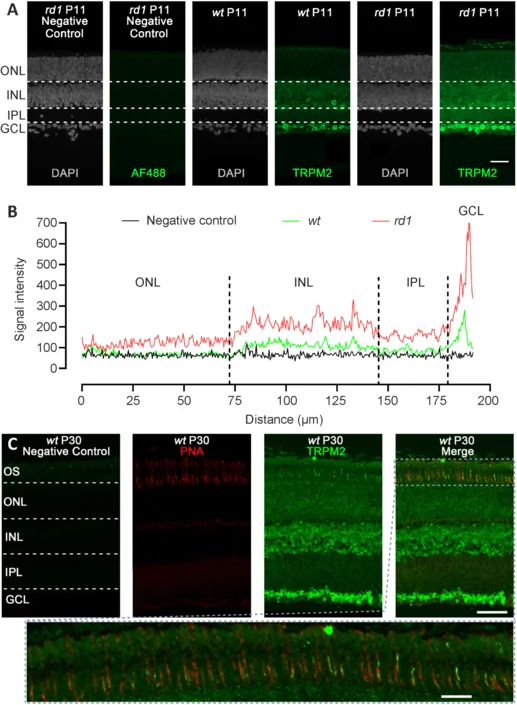
Retinal TRPM2 expression in *wt* and *rd1* retina. (A) Immunostaining for TRPM2 (green) in post-natal day (P) 11 *wt* and *rd1* retina. 4′,6-Diamidino-2-phenylindole (gray) was used as a nuclear counterstain. (B) Vertical localisation of TRPM2 signal in *wt* and *rd1* retina illustrated by an intensity distribution plot. Traces show average signal intensities from *wt* (*n* = 3) and *rd1* (*n* = 3) retina, the red trace indicates the *rd1* situation, green represents *wt*, black represents negative control. (C) Co-immunostaining for TRPM2 and the cone marker peanut agglutinin (PNA). TRPM2 was found to be partially co-localized with PNA in the outer segments of photoreceptors, indicating its expression in cones. Images are representative for at least three specimens and immunostainings. Scale bars: 50 µm. GCL: Ganglion cell layer; INL: inner nuclear layer; IPL: inner plexiform layer; ONL: outer nuclear layer; OS: outer segment; PNA: peanut agglutinin; rd1: retinal degeneration 1; TRPM2: transient receptor potential channel M2; wt: wild-type.

To assess such a possible association of TRPM2 expression with *rd1* degeneration, we first performed an immunostaining using an antibody directed against TRPM2. In the early post-natal retina, at P11, TRPM2 was found to be expressed in both *rd1* and wt retinas, notably in photoreceptor segments, inner nuclear layer, and in retinal ganglion cells (**Figure 1A**). However, at P11, the relative TRPM2 signal overall appeared to be stronger in *rd1* retinas than in wt, an observation confirmed by fluorescence intensity distribution plots (**Figure 1B**). In adult wt retina at P30, TRPM2 co-staining with the cone-specific marker PNA showed that TRPM2 was expressed in cone outer segments (**Figure 1C**). Taken together, these results illustrated the expression of TRPM2 protein in the retina, including in photoreceptors.

### Inhibition of transient receptor potential channel M2 changes the enzymatic activity of poly(ADP-ribose) polymerase

**Figure 2:**
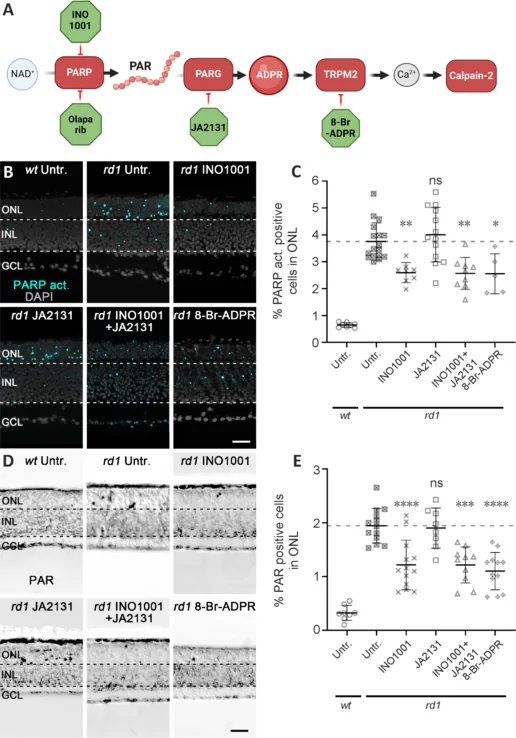
INO1001 and 8-Br-ADPR effectively reduce PARP activity and PAR generation. (A) Diagram illustrating how PARP-signaling may influence Ca^2+^-influx and calpain-2 activity. PARP, PARG, and TRPM2 were inhibited using INO1001 (PARP inhibitor), JA2131 (PARG inhibitor), and 8-Br-ADPR (TRPM2 blocker), respectively. (B) PARP activity assay (cyan) was performed in *rd1* and *wt* retinal explant cultures. 4′,6-Diamidino-2-phenylindole (gray) was used as nuclear counterstain. Untreated (Untr.) *rd1* and *wt* retina were compared with *rd1* retina treated with either INO1001, JA2131, INO1001 combined with JA2131, or 8-Br-ADPR. (C) Scatter plot showing percentage of PARP activity positive cells in the outer nuclear layer (ONL). Untr. *wt*: *n* = 8; Untr. *rd1*: *n* = 17; INO1001 *rd1*: *n* = 8; JA2131 *rd1*: *n* = 13; INO1001+JA2131 *rd1*: *n* = 9; 8-Br-ADPR *rd1*: *n* = 5. (D) PAR staining (black) was performed in *rd1* and *wt* retinal explant cultures. Scale bars: 50 µm. (E) Untreated *wt* and *rd1* retina were compared to drug-treated retina as in A. Untr. *wt*: *n* = 8; Untr. *rd1*: *n* = 13; INO1001 *rd1*: *n* = 13; JA2131 *rd1*: *n* = 8; INO1001 + JA2131 *rd1*: *n* = 10; 8-Br-ADPR *rd1*: *n* = 12. Statistical testing: One-way analysis of variance with Tukey’s multiple comparison *post hoc* test comparing *rd1* explant cultures. Error bars represent standard deviation. *P < 0.05, **P < 0.01, ***P < 0.001, ****P < 0.0001. ADPR: Mono adenosine diphosphate ribose (ADP-ribose); GCL: ganglion cell layer; INL: inner nuclear layer; NAD^+^: nicotinamide adenine dinucleotide; ns: not significant; ONL: outer nuclear layer; PAR: poly(ADP-ribose); PARG: poly(ADP-ribose) glycohydrolase; PARP: poly(ADP-ribose) polymerase; PARP act.: poly(ADP-ribose) polymerase activity; rd1: retinal degeneration 1; TRPM2: transient receptor potential channel M2; wt: wild-type.

The PARP enzyme uses NAD^+^ to generate PAR polymers, which in turn can be degraded by PARG to ADPR. ADPR monomers can activate TRPM2 channels, allowing for Ca^2+^-influx (**Figure 2A**). Calpains are sensitive to intracellular Ca^2+^ levels and among the various calpain isoforms, calpain-2 is activated by high, millimolar Ca^2+^ concentrations (Kumar and Stewart, 2025). Calpain activity and labeling of activated calpain-2 may therefore be used to indirectly determine intracellular Ca^2+^ overload in photoreceptors. To investigate the relationship between TRPM2 and PARP activity, as well as between TRPM2 and PARG activity during *rd1* degeneration, we used a range of different compounds to selectively inhibit these cellular targets. Along the putative PARP-signaling pathway (**Figure 2A**), treatments with the inhibitors INO1001, JA2131, and 8-Br-ADPR were used to inhibit either PARP, PARG, or TRPM2, respectively. The well-established PARP inhibitor Olaparib, which was previously tested in *rd1* retina (Yan et al., 2022), was used for additional controls. Dose response curves for INO1001, JA2131, and 8-Br-ADPR were generated to select appropriate treatment concentrations (**Additional Figure 1A–C**). An overview of the compounds used and their inhibitory capacities is shown in **Additional Figure 1** and **Additional Table 1**.

To confirm the efficacy of PARP inhibitors, we performed an *in situ* PARP activity assay and an immunostaining for PAR, i.e. a staining for the product of PARP activity. While PARP activity and PAR-positive cells were infrequent in wt retina when compared with *rd1* retina, treatment with INO1001 significantly reduced both PARP activity and PAR accumulation in the *rd1* ONL (**Figure 2B–E** and **Additional Table 2**; dose-response curve shown in **Additional Figure 1A**). However, the PARG inhibitor, JA2131, did not affect PARP activity and PAR in *rd1* ONL (**Figure 2B–E** and **Additional Table 2**). In contrast, PARP activity and PAR-positive cells in *rd1* ONL were significantly reduced after treatment with the combination of INO1001 and JA2131, as well as after single treatment (i.e. monotherapy) with 8-Br-ADPR (**Figure 2B–E** and **Additional Table 2**). Also, in rd1*Cngb1^−/−^ retinas, which lack functional cGMP-activated CNG-channels, both Olaparib and INO1001 significantly reduced PARP activity and PAR generation (**Additional Figure 2A–D**), suggesting that PARP activity was mostly independent of CNG-channel function. Overall, this data showed that both INO1001 and 8-Br-ADPR efficiently reduced PARP activity, while inhibition of PARG with JA2131 did not.

**Additional Table 2:** Quantification of calpain-2 activation, calpain activity, PARP activity, and PAR-positive cells in the ONL

		% Positive cells in ONL (mean±SD)	Sample (*n*)	Turkey's multiple comparison test	*P*-value
**PARP activity**	*wt* Unix.	0.65±0.84	8	*wt* Untr. *vs*. *rd1* Untr.	< 0.0001
	*rd1* Untr.	3.75±0.68	17		
	*rd1* INO1001	2.60±0.35	8	*rd1* INO1001 *vs*. *rd1* Untr.	< 0.01
	*rd1* JA2131	4.00±0.98	13	*rd1* JA2131 *vs*. *rd1* Untr.	> 0.05
	*rd1* INO1001+JA2131	2.57±0.56	9	*rd1* INO1001+JA2131 *vs*. *rd1* Untr.	< 0.01
	*rd1* 8-Br-ADPR	2.55±0.66	5	*rd1* 8-Br-ADPR *vs*. *rd1* Untr.	< 0.05
**PAR**	*wt* Untr.	0.31±0.14	8	*wt* Untr. *vs*. *rd1* Untr.	< 0.0001
	*rd1* Untr.	1.94±0.31	13		
	*rd1* INO1001	1.22±0.44	13	*rd1* INO1001 *vs*. *rd1* Untr.	< 0.001
	*rd1* JA2131	1.90±0.35	8	*rd1* JA2131 *vs*. *rd1* Untr.	> 0.05
	*rd1* INO1001+JA2131	1.22±0.32	10	*rd1* INO1001+JA2131 *vs*. *rd1* Untr.	< 0.01
	*rd1* 8-Br-ADPR	1.10±0.33	12	*rd1* 8-Br-ADPR *vs*. *rd1* Untr.	**<** 0.0001
**Calpain activity**	*wt* Untr.	0.72±0.10	8	*wt* Untr. *vs*. *rd1* Untr.	< 0.0001
	*rd1* Untr.	3.93±0.82	18		
	*rd1* INO1001	2.63±0.45	8	*rd1* INO1001 *vs*. *rd1* Untr.	< 0.01
	*rd1* JA2131	2.80±0.81	13	*rd1* JA2131 *vs*. *rd1* Untr.	< 0.01
	*rd1* INO1001+JA2131	2.63±0.45	8	*rd1* INO1001+JA2131 *vs*. *rd1* Untr.	< 0.01
	*rd1* 8-Br-ADPR	2.44±0.92	6	*rd1* 8-Br-ADPR *vs*. *rd1* Untr.	< 0.01
**Calpain-2**	*wt* Untr.	0.66±0.08	6	*wt* Untr. *vs*. *rd1* Untr.	< 0.0001
	*rd1* Untr.	3.29±0.42	17		
	*rdl* INO1001	2.20±0.59	13	*rdl* INO1001 *vs*. *rdl* Untr.	< 0.0001
	*rdl* JA2131	2.16±0.47	10	*rdl* JA2131 vs *rdl* Untr.	< 0.0001
	*rdl* INO1001+JA2131	2.27±0.43	10	*rdl* INO1001+JA2131 *vs*. *rdl* Untr.	< 0.0001
				*rdl* INO1001+JA2131 *vs*. *rdl* INO1001	> 0.05
				*rdl* INO1001+JA2131 *vs*. *rdl* JA2131	> 0.05
	*rdl* 8-Br-ADPR	2.20±0.44	12	*rdl* 8-Br-ADPR *vs*. *rdl* Untr.	< 0.0001

INO1001: PARP inhibitor; JA2131: PARP inhibitor. ONL: Outer nuclear layer; PAR: poly(ADP-ribose); PARP: poly(ADP-ribose) polymerase; *rdl:* retinal degeneration 1; Untr.: untreated.

### Poly(ADP-ribose) polymerase, poly(ADP-ribose) glycohydrolase, and transient receptor potential channel M2 regulate calpain activity and calpain-2 activation

**Figure 3:**
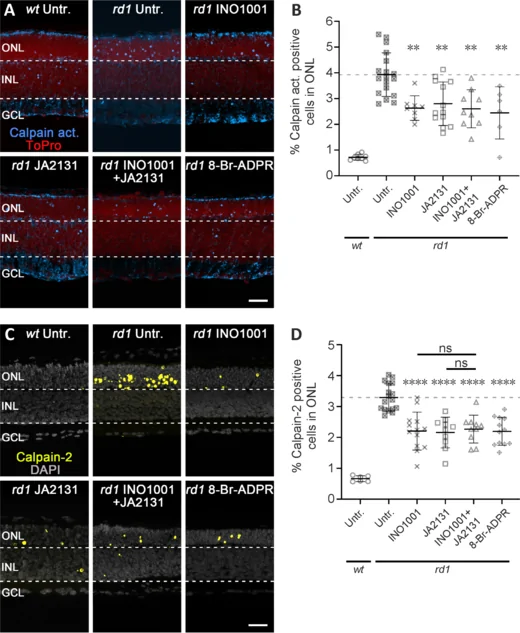
Inhibition of PARP-signaling decreases calpain activity and calpain-2 activation. (A) Calpain activity assay (blue) was performed on *wt* and *rd1* retinal explant cultures. ToPro (red) was used as a nuclear counterstain. Untreated (Untr.) *wt* and *rd1* retina were compared to retina treated with INO1001 (PARP inhibitor), JA2131 (PARG inhibitor), INO1001 combined with JA2131, and 8-Br-ADPR (TRPM2 blocker). Note the high number of cells displaying calpain activity in the ONL. Scale bar: 50 µm. (B) Scatter plot showing percent calpain activity positive cells in the ONL. Untr. *wt*: *n* = 8; Untr. *rd1*: *n* = 18; INO1001 *rd1*: *n* = 8; JA2131 *rd1*: *n* = 13; INO1001 + JA2131 *rd1*: *n* = 8; 8-Br-ADPR *rd1*: *n* = 6. (C) Immunostaining for activated calpain-2 (yellow) was performed using wt and rd1 retinal explant cultures. 4′,6-Diamidino-2-phenylindole (gray) was used as a nuclear counterstain. Untreated *wt* and *rd1* retina were compared to retina treated with INO1001, JA2131, 8-Br-ADPR, or a combination of INO1001 and JA2131. Scale bars: 50 µm. (D) Scatter plot showing the percentage of ONL cells displaying calpain-2 activation. Untr. *wt*: *n* = 6; Untr. *rd1*: *n* = 17; INO1001 *rd1*: *n* = 13; JA2131 *rd1*: *n* = 10; INO1001 + JA2131 *rd1*: *n* = 10; 8-Br-ADPR *rd1*: *n* = 12. Statistical testing: One-way analysis of variance and Tukey’s multiple comparison *post hoc* test; significance levels: **P < 0.01, ****P < 0.0001; error bars represent standard deviation. calpain act.: Calpain activity; GCL: ganglion cell layer; HDAC: histone-deacetylases; INL: inner nuclear layer; ns: not significant; ONL: outer nuclear layer; PARP: poly(ADP-ribose) polymerase; rd1: retinal degeneration 1; wt: wild-type.

In a recent study, we found the activity of Ca^2+^-dependent calpain-type proteases to likely be controlled by PARP activity (Yan et al., 2024). To further study how PARP, PARG, and TRPM2 inhibition impacted calpain activity, we first used a general in situ assay that can resolve enzymatic activity of calpains in individual retinal cells (Belhadj et al., 2022). Calpain activity was rather low in wt retina when compared with *rd1* retina (**Figure 3A** and **Additional Table 2C**). Treatments with INO1001, JA2131, INO1001 combined with JA2131, and 8-Br-ADPR all led to a significant reduction of the numbers of ONL cells showing calpain activity (**Figure 3B** and **Additional Table 2D**; dose-response curve shown in **Additional Figure 1A–C**). Remarkably, neither Olaparib nor INO1001 treatment could reduce calpain activity in rd1*Cngb1^−/−^ photoreceptors, i.e. in photoreceptors in which CNG-channels are dysfunctional (**Additional Figure 3A** and **B**). Since calpain activity in *rd1* single-mutant retina was significantly higher than in the rd1*Cngb1^−/−^ situation, this outcome suggested that calpain activation was in part caused by CNG-channel independent Ca^2+^-influx. Moreover, in the rd1*Cngb1^−/−^ double mutant retina calpain activity was apparently triggered independent of PARP.

Among the calpain-type proteases, calpain-2 appears to be the isoform that is most important for neurodegenerative processes (Baudry and Bi, 2025) and immunolabeling for activated calpain-2 may provide a snapshot that allows researchers to infer changes in intracellular Ca^2+^ levels. Similar to total calpain activity, the number of cells in the ONL of wt retina displaying calpain-2 activation was rather low when compared with *rd1* retina (**Figure 3C** and **Additional Table 2**). In *rd1* retina, the drugs INO1001, JA2131, and ADPR, individually, significantly reduced calpain-2 activation (**Figure 3D** and **Additional Table 2**). A combined inhibition of PARP and PARG with INO1001 and JA2131 did not produce an additional synergistic effect (**Figure 3D** and **Additional Table 2**), indicating that both enzymes belonged to the same calpain-2 activating pathway. However, similar to what was seen for general calpain activity, in rd1*Cngb1^−/−^ double-mutant retina the PARP inhibitors Olaparib and INO1001 could not reduce calpain-2 activation (**Additional Figure 3C** and **D**).

### Inhibition of Poly(ADP-ribose) polymerase, poly(ADP-ribose) glycohydrolase, and transient receptor potential channel M2 alters histone deacetylase activity and reduces cell death

**Figure 4:**
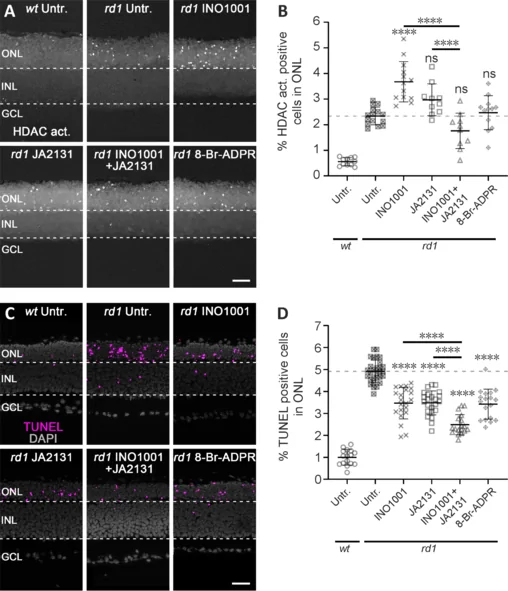
HDAC activity, and photoreceptor cell death after the inhibition of PARP-signaling. (A) HDAC activity assay (white) was performed in *rd1* and *wt* retinal explant cultures. Untreated *rd1* and *wt* retina were compared with *rd1* retina treated with either INO1001 (PARP inhibitor), JA2131 (PARG inhibitor), INO1001 + JA2131, or 8-Br-ADPR (TRPM2 blocker). Scale bar: 50 µm. (B) Scatter plot showing percent displaying HDAC activity. Untr. *wt*: *n* = 11; Untr. *rd1*: *n* = 15; INO1001 *rd1*: *n* = 13; JA2131 *rd1*: *n* = 10; INO1001 + JA2131 *rd1*: *n* = 10; 8-Br-ADPR *rd1*: *n* = 12. (C) TUNEL assay labeling dying cells (magenta) in *rd1* and *wt* retinal explant cultures. 4′,6-Diamidino-2-phenylindole (gray) was used as a nuclear counterstain. Untreated *wt* and *rd1* retinas were compared to retinas treated with compounds as in A. Untr. *wt*: *n* = 16; Untr. *rd1*: *n* = 29; INO1001 *rd1*: *n* = 21; JA2131 *rd1*: *n* = 23; INO1001 + JA2131 *rd1*: *n* = 18; 8-Br-ADPR *rd1*: 18. Scale bar: 50 µm. (D) Scatter plot showing the percentage of TUNEL-positive cells. Note the significant elevation of HDAC activity caused by INO1001 alone and the synergistic protective effect on TUNEL-positive cells when INO1001 and JA2131 treatments were combined. Statistical testing: One-way analysis of variance and Tukey’s multiple comparison *post hoc* test; ****P < 0.0001; error bars represent SD. GCL: Ganglion cell layer; HDAC: histone deacetylase; HDAC act.: histone deacetylase activity; INL: inner nuclear layer; ONL: outer nuclear layer; rd1: retinal degeneration 1; TUNEL: terminal deoxynucleotidyl transferase dUTP nick end labeling; wt: wild-type.

The activity of HDAC was previously suggested to contribute to photoreceptor degeneration (Dong et al., 2023a, b). We investigated a possible link to PARP-, PARG-, and TRPM2-related signaling using a general HDAC *in situ* activity assay that non-selectively detects activity of isoforms belonging to all four classes of HDACs. In *rd1* retina, the number of HDAC activity positive cells in the ONL was higher compared to wt retina (**Figure 4A** and **Additional Table 3**). Unexpectedly, INO1001 significantly increased overall HDAC activity in *rd1* ONL, while treatment with JA2131, INO1001 + JA2131, and 8-Br-ADPR did not change the numbers of HDAC activity positive cells, as compared to untreated *rd1* cultures (**Figure 4B** and **Additional Table 3**). When compared to the monotherapies of INO1001 and JA2131, combined treatment with INO1001 + JA2131 significantly reduced HDAC activity (**Figure 4B** and **Additional Table 3**).

**Additional Table 3:** Quantification of HDAC activity and TUNEL-positive, dying cells in the ONL

		% Positive cells in ONL (mean±SD)	Sample (*n*)	Turkey's multiple comparison test	*P*-value
**HDAC activity**	*wt* Untr.	0.55±0.15	11	*wt* Untr. *vs*. *rdl* Untr.	< 0.0001
	*rdl* Untr.	2.34±0.33	15		
	*rdl* INO1001	3.70±0.76	13	*rdl* INO1001 *vs*. *rdl* Untr.	< 0.0001
	*rdl* JA2131	2.97±0.59	10	*rdl* JA2131 *vs*. *rdl* Untr.	> 0.05
	*rdl* INO1001+JA2131	1.76±0.66	10	*rdl* INO1001+JA2131 *vs*. *rdl* Untr.	> 0.05
				*rdl* INO1001+JA2131 *vs*. *rdl* INO1001.	< 0.0001
				*rdl* INO1001+JA2131 *vs*. *rdl* JA2131.	< 0.0001
	*rdl* 8-Br-ADPR	2.47±0.64	12	*rdl* 8-Br-ADPR *vs*. *rdl* Untr.	> 0.05
**TUNEL**	*wt* Untr.	1.00±0.35	16	*wt* Untr. *vs*. *rdl* Untr.	< 0.0001
	*rdl* Untr.	4.91±0.51	29		
	*rdl* INO1001	3.47±0.70	21	*rdl* INO1001 *vs*. *rdl* Untr.	< 0.0001
	*rdl* JA2131	3.48±0.53	23	*rdl* JA2131 *vs*. *rdl* Untr.	< 0.0001
	*rdl* INO1001+JA2131	2.49±0.45	18	*rdl* INO1001+JA2131 *vs*. *rdl* Untr.	< 0.0001
	*rdl* 8-Br-ADPR	3.42±0.66	18	*rdl* 8-Br-ADPR *vs*. *rdl* Untr.	< 0.0001

INO1001: Poly(ADP-ribose) polymerase inhibitor; JA2131: poly(ADP-ribose) glycohydrolase inhibitor. HDAC: Histone-deacetylases; ONL: outer nuclear layer; *rdl:* retinal degeneration 1; TUNEL: terminal deoxynucleotidyl transferase dUTP nick end labelling; Untr.: untreated.

**Figure 5:**
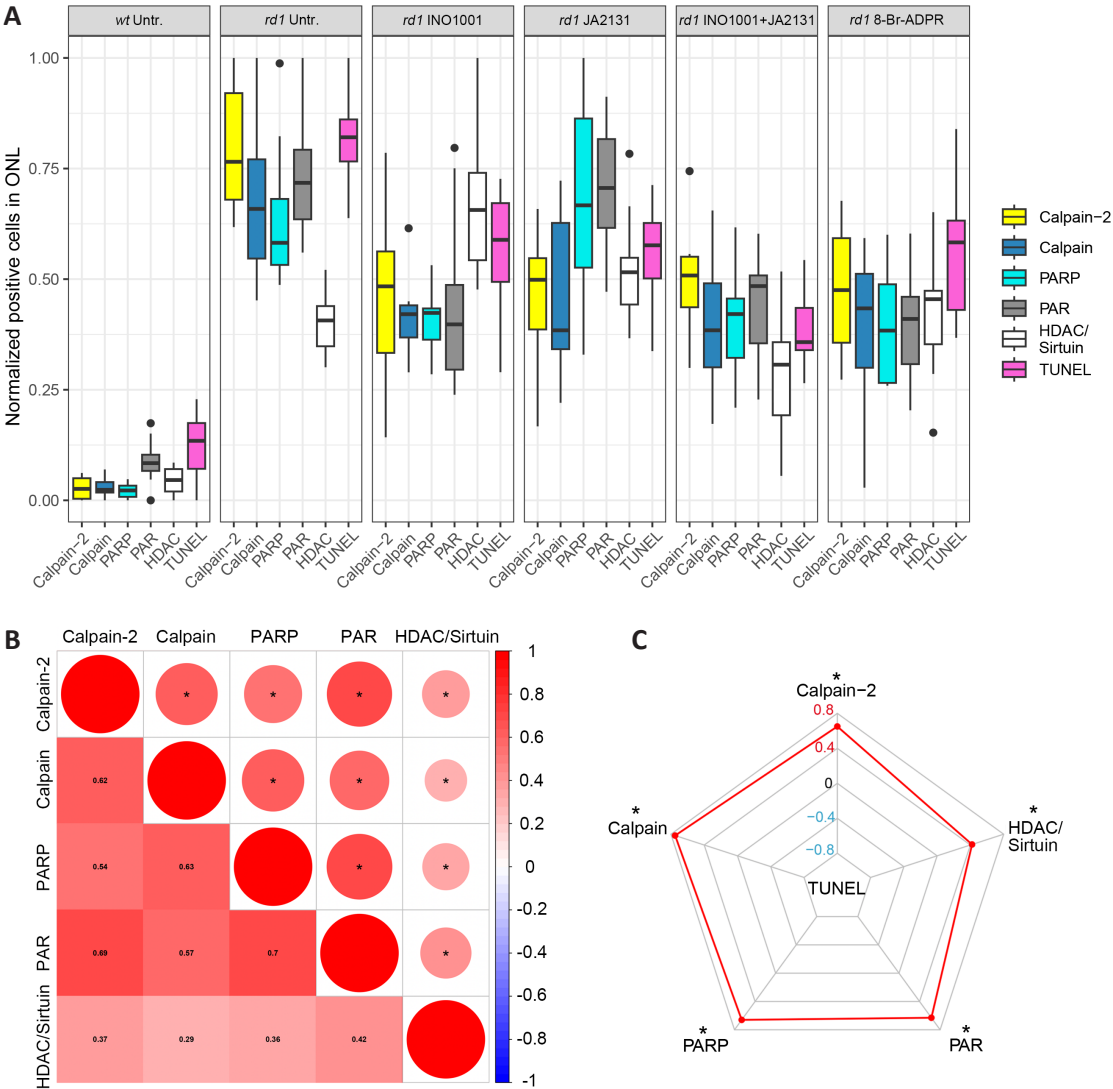
Enzymatic signatures for rd1 photoreceptor degeneration and Spearman analysis. (A) Comparison of enzymatic markers across different experimental treatments. Normalized cell numbers positive for TUNEL (magenta), calpain activity (calpain, blue), activated calpain-2 (yellow), PARP activity (PARP, cyan), PAR (black), and HDAC/Sirtuin activity (white). (B) Spearman analysis of enzymatic markers in photoreceptors (calpain activity, calpain-2, PARP activity, PAR, HDAC). The asterisks in circles show statistical significance, numbers in squares present the *R*^2^. (C) Radar plot for Spearman analysis revealing the correlation between different enzymatic markers and the TUNEL assay. Note the comparatively weak association between HDAC activity and cell death (TUNEL). HDAC: Histone deacetylase; PAR: poly(ADP-ribose); PARP: poly(ADP-ribose) polymerase; rd1: retinal degeneration 1; TUNEL: terminal deoxynucleotidyl transferase dUTP nick end labeling; wt: wild-type.

To confirm the link between PARP-signaling and photoreceptor degeneration, the TUNEL assay was used to quantify the numbers of dying cells in the ONL. In wt retinal cultures, the numbers of TUNEL-positive cells in the ONL was significantly lower than in its *rd1* counterpart (**Figure 4C** and **Additional Table 3**). Both INO1001 and JA2131 significantly decreased photoreceptor cell death in *rd1* cultures. Remarkably, INO1001 combined with JA2131 reduced the numbers of TUNEL-positive cells in the ONL even further, when compared with untreated *rd1*, and monotherapies of INO1001 or JA2131 (**Figure 4D** and **Additional Table 3**). This was surprising since PARP and PARG would be expected to take part in the same pathway (Harrision et al., 2020). To confirm the synergistic effect observed, we combined JA2131 with Olaparib treatment in *rd1* retinal cultures. However, this combination failed to show the synergistic effect seen with INO1001 and JA2131 (**Additional Figure 4A** and **B**). Moreover, in rd1*Cngb1^−/−^ double-mutant mice, INO1001 treatment reduced photoreceptor cell death when Olaparib did not (**Additional Figure 5A** and **B**), strongly suggesting a difference in the mode of action or the targets for these two compounds.

### Enzyme activity patterns are altered by interventions targeting poly(ADP-ribose) polymerase-signaling

To compare the various enzymatic and cell death markers with each other, we normalized the experimental datasets by linear scaling, such that the lowest values were set to zero while the highest values were set to one. In wt retinas, all enzyme activity and cell death markers were generally low when compared with the *rd1* untreated groups (**Figure 5A**). In untreated *rd1*, the number of ONL cells showing HDAC activity was lower than calpain-2 activation, calpain activity, PARP activity, and PAR-positive cells. Photoreceptor cell death was significantly reduced by monotherapies of INO1001, JA2131, and 8-Br-ADPR. However, INO1001 led to relatively high HDAC activity, while JA2131 caused high PARP activity and PAR accumulation compared to other markers (**Figure 5A**). The combined treatment with INO1001 and JA2131 synergistically reduced photoreceptor degeneration and all related markers, relative to monotherapies with INO1001 or JA2131. Although also 8-Br-ADPR led to relatively low levels for all enzyme activity markers, it did not decrease cell death as much as the combination therapy of INO1001 and JA2131 (**Figure 5A**). The relative increase in overall HDAC activity caused by INO1001, but not by any of the other drug treatments, was remarkable and could indicate an activation of Sirtuin-type HDACs.

To better understand these activity patterns triggered by inhibition of PARP, PARG, or TRPM2, we performed a Spearman analysis (**Figure 5B**), including all datasets. General calpain activity, calpain-2 activation, PARP activity, PAR, and HDAC were positively correlated with each other (*P* < 0.05, *R*^2^ values: calpain-2-calpain, 0.62; PARP-calpain-2, 0.54; PAR-calpain-2, 0.69; HDAC/sirtuin-calpain-2, 0.37; calpain-PARP, 0.63; calpain-PAR, 0.57; calpain-HDAC/sirtuin, 0.29; PARP-PAR, 0.7; PARP-HDAC/sirtuin, 0.36; PAR-HDAC/sirtuin, 0.42). A radar plot was used to show the relationship of cell death and enzymatic signatures after Spearman analysis (**Figure 5C**). In the *rd1* ONL, this illustrated a positive correlation between cell death (TUNEL-positive cells) calpain activity, calpain-2 activation, PARP activity, and PAR, but less so with HDAC activity (*P* < 0.05, *R*^2^ values: TUNEL-calpain-2, 0.65; TUNEL-HDAC, 0.42; TUNEL-calpain, 0.75; TUNEL-PARP, 0.66; PAR, 0.63; **Figure 5C**). Taken together, these analyses indicated that calpain activity, calpain-2, PARP, PAR, and HDAC activity were all connected to cell death. Then again, the association of HDAC activity with cell death was not as strong as for the other four markers, suggesting that among the various HDAC isoforms/classes there could also be such that might have protective effects.

## Discussion

In the *rd1* mouse retina, we previously found PARP to regulate calpain activity, promoting photoreceptor degeneration (Yan et al., 2022). However, the mechanism of this PARP-dependent regulation of calpain was still unclear. Our present work proposes that PARP controls calpain activity likely through PARG-induced ADPR formation and TRPM2-associated Ca^2+^ influx. The blockage of either PARP, PARG, or TRPM2 was found to be neuroprotective. Notably, the combined therapy with the PARP inhibitor INO1001 and the PARG inhibitor JA2131 produced a synergistic therapeutic effect in *rd1* retina. These data suggest not only PARP, but also PARG and TRPM2 as therapeutic targets for the treatment of IRD. Overall, our study indicates PARP-dependent Ca^2+^ regulation to be detrimental to photoreceptor viability.

### Poly(ADP-ribose) polymerase-dependent Ca^2+^ overload in photoreceptors

**Figure 6:**
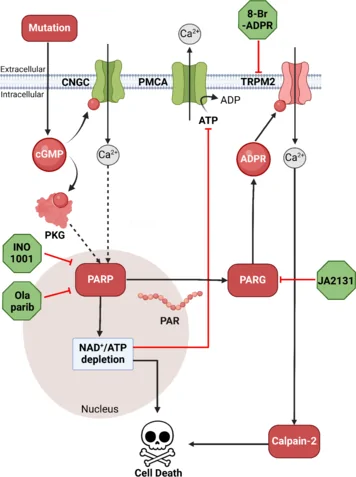
PARP-signaling and experimental interventions in cGMP-dependent *rd1* degeneration. In *rd1* photoreceptors, the Pde6b mutation induces cyclic guanosine monophosphate (cGMP) accumulation, which activates both the cyclic-nucleotide-gated channel (CNGC) and protein kinase G (PKG). Via yet unknown mechanisms, excessive PKG activity may result in DNA damage, which likely leads to poly(ADP-ribose)-polymerase (PARP) activation. On one hand, poly(ADP-ribose) (PAR) generated by PARP may be cleaved by poly(ADP-ribose) glycohydrolase (PARG) into ADP-ribose (ADPR). ADPR can directly open transient receptor potential channel M2 (TRPM2), leading to Ca^2+^ influx. On the other hand, PARP activation may entail a depletion of nicotinamide adenine dinucleotide (NAD^+^) and adenosine triphosphate (ATP). Since ATP is required for plasma membrane Ca^2+^ ATPase (PMCA)-mediated Ca^2+^ extrusion, loss of ATP and TRPM2 opening, in concert, lead to increased intracellular Ca^2+^ levels and excessive activation of calpain-type proteases. Eventually, the activities of calpain-2, PARP, and intracellular Ca^2+^ overload promote photoreceptor cell death.

In the present study, we investigated the roles of PARP, PARG, and TRPM2 in photoreceptor cell death (**Figure 6**). PARP activity is connected to the *rd1* mutation in the *Pde6b* gene, likely via increased photoreceptor cGMP-levels (**Figure 6**; Power et al., 2020; Yan et al., 2024). Previously, we found the PARP inhibitor Olaparib to reduce the enzymatic activity of Ca^2+^-dependent calpain-type proteases and to slow down the progression of *rd1* photoreceptor degeneration (Yan et al., 2022). Here, we found the alternative PARP inhibitor, INO1001, to show similar neuroprotective effects, consolidating the idea that PARP plays an important role in photoreceptor cell death. PARP catalyses the covalent attachment of PAR polymers onto itself by using NAD^+^ as a donor of ADPR units (Mao and Zhang, 2022). Covalently attached PAR can be hydrolyzed to free PAR polymers or ADPR monomers by PARG, which possesses both endoglycosidic and exoglycosidic activities, and by PAR hydrolase (ARH3), which also has PARG activity (Luo and Kraus, 2012). Overactivation of PARP may lead to NAD^+^ depletion (Yan et al., 2021), which will decrease photoreceptors’ capacity to produce ATP. At the same time, increased generation of ADPR by PARP and PARG, and subsequent activation of TRPM2 will drive up Ca^2+^ influx (AlAhmad et al., 2025; Shitaw et al., 2025). Consequently, ATP consumption for Ca^2+^ extrusion via the ATP-driven plasma membrane Ca^2+^-ATPase rises (**Figure 6**). Overall, a PARP-dependent decreased ATP production, combined with increased ATP consumption, is likely to be strongly detrimental for photoreceptor cell viability (Ames, 1992).

When we used the PARG inhibitor JA2131 (Houl et al., 2019) on *rd1* retinal explant cultures, calpain activity and calpain-2 activation were significantly reduced, suggesting that ADPR changes induced by PARG inhibition also affected intracellular Ca^2+^ levels in *rd1* retina. The photoreceptor protective effect of PARG inhibition is in line with an earlier study which showed that a genetic deletion of PARG was neuroprotective (Sahaboglu et al., 2014). The fact that both PARP and PARG inhibition reduced calpain activity – and by extension Ca^2+^-influx – hinted at TRPM2 as a likely candidate for causing Ca^2+^ overload in *rd1* photoreceptors. This idea was supported by the use of the TRPM2 ion channel antagonist 8-Br-ADPR (Rah et al., 2015), which significantly reduced the enzymatic activity of calpain. TRPM2 inhibition also decreased PARP activity, suggesting a possible Ca^2+^-mediated feedback loop that regulated PARP activity. This is consistent with a recent study which found that Ca^2+^-levels regulated PARP activity and promoted photoreceptor degeneration (Yan et al., 2024). It seems likely that an elevated intracellular Ca^2+^ concentration leads to activation of calpain type proteases. Among these, calpain-2 – activated by millimolar Ca^2+^-levels – is likely to be particularly detrimental to cell survival, while calpain-1 – activated by much lower micromolar Ca^2+^-levels – may have a protective role (Baudry and Bi, 2025). Future studies using cell-type specific, conditional genetic manipulations of calpain-1 and -2 may yield further insights into the interplay of these calpain isoforms with intracellular Ca^2+^ and PARP activity. Taken together, our data indicate that in cGMP-induced photoreceptor degeneration PARP regulates Ca^2+^-dependent calpain activity through ADPR-mediated activation of TRPM2.

### Efficacy of INO1001 in hereditary retinal degeneration

PARP activity was previously connected to photoreceptor degeneration (Yan et al., 2022) and different PARP inhibitors, including Olaparib and PJ34, have shown neuroprotective effects in various IRD models (Sahaboglu et al., 2017; Yan et al., 2022). Compared to these drugs, the PARP inhibitor INO1001 (Brock et al., 2004) used here showed an improved efficacy in the *rd1* mouse model. Surprisingly, the combination of INO1001 with the PARG inhibitor JA2131 showed a synergistic effect in reducing photoreceptor cell death without further reducing PARP and calpain activity, when compared to individual treatments with either INO1001 or JA2131. This suggests that the activities of PARP and calpain may be partially independent of the execution of photoreceptor cell death.

To further investigate this effect, we combined Olaparib with JA2131, which, however, did not synergistically decrease photoreceptor cell death in *rd1*. These results indicate that the mode of action or the targets of INO1001 could be different from Olaparib. In support of this view, a recent article found that INO1001 and Olaparib differ significantly in their inhibitory profiles. Both compounds potently inhibit the PARP-1 isoform, yet INO1001 inhibits PARP-2 only weakly, while Olaparib also strongly blocks this isoform (Pires et al., 2024). Whereas PARP-1 likely accounts for more than 90% of total cellular PARP activity (Schiewer and Knudsen, 2014), PARP-2 may, under certain conditions, have neuroprotective effects (Kofler et al., 2006). Moreover, PARP-2 activity may be specifically enhanced by PAR chains (Chen et al., 2018), an effect that is counteracted by the activity of PARG. In a situation where PARP-1 is efficiently inhibited by INO1001 but PARP-2 remains active, the additional inhibition of PARG by JA2131 may drive a selective increase in PARP-2 activity. It is therefore plausible to think that the synergistic beneficial effect seen in the combined treatment with INO1001 and JA2131 may depend on such an increase in PARP-2 activity. This could also, in part, explain why a synergistic effect of INO1001 and JA2131 was seen in the cell death assay but not in general PARP activity. However, at this point, we cannot exclude the possibility that INO1001 hits targets entirely unrelated to PARP, and that these may instead be responsible for the observed beneficial effects. Even though such a coincidence may seem improbable, in this case, the synergistic effect of INO1001 and JA2131 would be related to two independent cell death-promoting pathways.

Besides, in the rd1*Cngb1^−/−^ retina, which lacks functional CNG-channels (Paquet-Durand et al., 2011), Olaparib failed to show a protective effect, while INO1001 significantly reduced photoreceptor cell death also in the double-mutant retina. This outcome likewise points to different modes of action for Olaparib and INO1001 and could furthermore indicate that CNG-channels may be more directly linked to PARP activity, perhaps because of compartmentalization and specific structural organization in the photoreceptor outer segments.

### Relationship between poly(ADP-ribose) polymerase and histone deacetylase activity

The HDAC family consists of 18 different mammalian isoforms divided into four classes based on their sequence similarity to yeast counterparts (Renzini et al., 2022). Depending on the mechanism of deacetylation of lysine residues on protein substrates, HDACs are divided into zinc-dependent (Class I, II, and IV) and NAD^+^-dependent (Class III) isoforms. The latter group is commonly referred to as Sirtuins or Sirtuin-type HDACs (Curcio et al., 2024). Zinc-dependent HDACs were found to promote photoreceptor degeneration in the *rd1* mouse model for IRD (Dong et al., 2023b), even though within this group of HDACs, there may also be individual isoforms with neuroprotective properties (Chen and Cepko, 2009). For NAD^+^-dependent Sirtuin-type HDACs an overall neuroprotective role appears very likely (Wu et al., 2022).

Since PARP is a major consumer of NAD^+^ and since Sirtuins are very sensitive to changes in NAD^+^-levels (Cantó and Auwerx, 2012; Cohen, 2020), Sirtuin activity is often inversely correlated to PARP activity (Chung and Joe, 2014). In our study, administration of INO1001 strongly and significantly increased HDAC activity in photoreceptors, without further increasing cell death. This hints at the possibility that the increased levels of HDAC activity seen with INO1001 treatment could refer to increased Sirtuin activity. A possible explanation for this effect might be that PARP inhibition and the resultant decrease in NAD^+^ consumption had allowed for Sirtuins to become (or remain) active. Furthermore, other studies have found links between PARP and Sirtuins, notably activation of the Sirtuin-1 isoform (Bai et al., 2011; Chung and Joe, 2014), supporting a close relationship between PARP and Sirtuin-type HDACs. Curiously, an increase of HDAC/Sirtuin activity was not seen after treatment of *rd1* explant cultures with the PARP inhibitor Olaparib (Dong et al., 2023b), again pointing at differences in the mode of action between INO1001 and Olaparib. Future studies may address these differences in drug action and their possible root causes in, for instance, different drug targets.

The following limitations should be noted for this study: The interventional experiments were all performed in organotypic retinal explant cultures under entirely controlled *in vitro* conditions. While unlikely based on ample previous experience with this culture system (e.g., Belhadj et al., 2020), it is still possible that the retina and its photoreceptors *in vivo* might behave differently, for instance, in terms of expression of PARP or TRPM2. Moreover, many of the conclusions drawn from the interventional studies are based on the expected specificities of the drugs used. Even though all drugs used have been extensively validated previously, we cannot exclude the possibility that some of the results obtained here may, in fact, have been caused by off-target effects of the drugs employed (*cf.* data obtained with INO1001 *versus* Olaparib). Future studies using genetic manipulations, for instance, a conditional knock-out of TRPM2 in rod photoreceptors, may validate and confirm our present data.

In conclusion, in IRD, the activities of calpain and PARP are both closely associated with photoreceptor degeneration. Our results now demonstrate that in cGMP-induced photoreceptor degeneration, PARP regulates calpain activity, likely via ADPR and TRPM2 induced Ca^2+^ influx. We found that not only PARP activity, but likewise the activities of PARG and TRPM2 can cause photoreceptor degeneration. Expanding this research to also include genetic modulation of PARP-signaling could further refine these therapeutic targets. Our study also shows that while the PARP inhibitor Olaparib potently reduced general PARP activity, INO1001 presented an overall better therapeutic efficacy in both *rd1* and rd1*Cngb1^−/−^ retina. Corresponding *in vivo* studies in IRD animal models, using appropriate delivery vehicles and administration routes, may validate the neuroprotective effects observed here and could facilitate future clinical translation. Taken together, these results emphasize the importance of PARP and its associated pathways as attractive targets for future therapeutic interventions in IRD.

## Additional files:

***Additional Figure 1:*** *Dose-response curves for INO1001, JA2131 (PARG inhibitor), and 8-Br-ADPR (TRPM2 blocker).*

Additional Figure 1 Dose-response curves for INO1001, JA2131 (PARG inhibitor), and 8-Br-ADPR (TRPM2 blocker).
(A) Different concentrations of INO1001 (PARP inhibitor) were tested in *rd1* mouse retinal explant cultures. In the ONL, at concentrations of 0.1 μM and 1 μM, INO1001 significantly decreased the numbers of ONL cells displaying calpain activity, PARP activity, and cell death (TUNEL assay). 0 μM *rd1*: *n* = 5; 0.1 μM *rd1*: *n* = 3; 1 μM *rd1*: *n* = 3; 10 μM *rd1*: *n* = 3. (B) Different concentrations of JA2131 tested in *rd1* explant cultures. In the ONL, at concentrations of 0.5, 5, and 25 μM, JA2131 significantly reduced ONL calpain activity, and cell death, as assessed by the TUNEL assay. 0 μM *rd1*: 6; 0.5 μM *rd1*: 3; 5 μM *rd1*: *n* = 3; 25 μM *rd1*: *n* = 3; 50 μM *rd1*: *n* = 3. (C) Dose-response for 8-Br-ADPR in *rd1* explant cultures. 50 μM and 100 μM 8-Br-ADPR significantly reduced calpain activity, PARP activity, and cell death (TUNEL) in the ONL. 0 μM *rd1*: 5; 25 μM *rd1*: *n* = 3; 50μM *rd1*: *n* = 3; 100 μM *rd1*: *n* = 3. Statistical testing: One-way analysis of variance with Tukey’s multiple comparison *post hoc* test comparing *rd1* explant cultures. Significance levels: ^*^P < 0.05. Error bars represent standard deviation. ONL: Outer nuclear layer; PARP: poly(ADP-ribose) polymerase; *rd1*: retinal degeneration 1; TUNEL: terminal deoxynucleotidyl transferase dUTP nick end labeling.

***Additional Figure 2:*** Effect of PARP inhibitors on rd1*Cngb1^*−/−*^ *retinal explant cultures.*

Additional Figure 2 Effect of PARP inhibitors on rd1^*^Cngb1^-/-^ retinal explant cultures.
(A) PARP activity assay (cyan) was performed in *rd1* and *wt* retinal explant cultures. 4ʹ,6-Diamidino-2-phenylindole (gray) was used as a nuclear counterstain. Untreated (Untr.) rd1^*^Cngb1^-/-^ and *wt* retina were compared to rd1^*^Cngb1^-/-^ retina treated with either INO1001 or Olaparib. (B) Scatter plot showing the percentage of PARP activity positive cells in the outer nuclear layer (ONL). Untr. *wt*: *n* = 7; Untr. rd1^*^Cngb1^-/-^: *n* = 11; INO1001 rd1^*^Cngb1^-/-^: *n* = 7; Olaparib rd1^*^Cngb1^-/-^: *n* = 9. (C) PAR staining (black) was performed in rd1^*^Cngb1^-/-^ and *wt* retinal explant cultures. Scale bars: 50 μm. (D) Untreated *wt* and rd1^*^Cngb1^-/-^ retina were compared to drug-treated retina as in A. Untr. *wt*: *n* = 9; Untr. rd1^*^Cngb1^-/-^: *n* = 20; INO1001 rd1^*^Cngb1^-/-^: *n* = 7; Olaparib rd1^*^Cngb1^-/-^: *n* = 14. Statistical testing: one-way analysis of variance with Tukey’s multiple comparison *post hoc* test performed between *rd1* explant cultures. Error bars represent standard deviation; ^****^P < 0.0001. GCL: Ganglion cell layer; INL: inner nuclear layer; ONL: outer nuclear layer; PARP act.: poly(ADP-ribose) polymerase activity; PAR: Poly(ADP-ribose); rd1^*^Cngb1^-/-^: retinal degeneration 1 cross breed Cngb1 knock-out; *wt*: wild-type.

***Additional Figure 3:*** In rd1*Cngb1^*−/−*^ *retinal explant cultures PARP inhibitors do not affect calpain activity or calpain-2 activation.*

Additional Figure 3 In rd1^*^Cngb1^-/-^ retinal explant cultures PARP inhibitors do not affect calpain activity or calpain-2 activation.
(A) Calpain activity assay (blue) was performed on wt and rd1^*^Cngb1^-/-^ retinal explant cultures. ToPro (red) was used as a nuclear counterstain. Untreated (Untr.) *wt* and rd1^*^Cngb1^-/-^ retina were compared to retina treated with INO1001 and Olaparib. (B) Scatter plot showing percent calpain activity positive cells in the ONL. Untr. *wt*: *n* = 8; Untr. rd1^*^Cngb1^-/-^: *n* = 11; INO1001 rd1^*^Cngb1^-/-^: *n* = 6; Olaparib rd1^*^Cngb1^-/-^: *n* = 9. (C) Immunostaining for activated calpain-2 (yellow) was performed using *wt* and rd1^*^Cngb1^-/-^ retinal explant cultures. 4ʹ,6-Diamidino-2-phenylindole (gray) was used as a nuclear counterstain. Untreated *wt* and rd1^*^Cngb1^-/-^ retina were compared to retina treated with INO1001 and Olaparib. Scale bars: 50 μm. (D) Scatter plot showing the percentage of ONL cells displaying calpain-2 activation. Untr. *wt*: *n* = 9; Untr. rd1^*^Cngb1^-/-^: *n* = 18; INO1001 rd1^*^Cngb1^-/-^: *n* = 7; Olaparib rd1^*^Cngb1^-/-^: *n* = 10. Statistical testing: one-way analysis of variance with Tukey’s multiple comparison post hoc test performed between rd1 explant cultures. Error bars represent standard deviation. calpain act.: Calpain activity; GCL: ganglion cell layer; INL: inner nuclear layer; ns: not significant; ONL: outer nuclear layer; rd1^*^Cngb1^-/-^: retinal degeneration 1 cross breed Cngb1 knock-out; *wt*: wild-type.

***Additional Figure 4:*** *Inhibition of PARP/PARG reduces rd1 photoreceptor cell death.*

Additional Figure 4 Inhibition of PARP/PARG reduces rd1 photoreceptor cell death.
(A) TUNEL assay labeling dying cells (magenta) in *rd1* retinal explant cultures. 4ʹ,6-Diamidino-2-phenylindole (gray) was used as a nuclear counterstain. *rd1* untreated retina treated *rd1* retina were compared to *rd1* retina treated with INO1001 (PARP inhibitor), Olaparib, JA2131 (PARG inhibitor), INO1001 + JA2131, and Olaparib + JA2131. Scale bar: 50 μm. (B) Scatter plot showing percent displaying TUNEL-positive cells. Note the synergistic effect of TUNEL-positive cells triggered by INO1001 + JA2131, but not by Olaparib + JA2131. Untr. *rd1*: n=29; INO1001 *rd1*: *n* = 21; Olaparib *rd1*: *n* = 13; JA2131 *rd1*: *n* = 23; INO1001+JA2131 *rd1*: *n* = 18; Olaparib + JA2131 *rd1*: *n* = 8. Statistical testing: one-way analysis of variance with Tukey’s multiple comparison *post hoc* test performed between *rd1* explant cultures. Error bars represent standard deviation; ^***^P < 0.001, ^****^P < 0.0001. GCL: Ganglion cell layer; INL: inner nuclear layer; ONL: outer nuclear layer; PARG: poly(ADP-ribose) glycohydrolase; PARP-2: poly(ADP-ribose) polymerase-2; *rd1*: retinal degeneration 1; TUNEL: terminal deoxynucleotidyl transferase dUTP nick end labeling.

***Additional Figure 5:*** The poly(ADP-ribose) polymerase inhibitor INO1001 reduces rd1*Cngb1^*−/−*^ *cell death, when Olaparib does not.*

Additional Figure 5 The poly(ADP-ribose) polymerase inhibitor INO1001 reduces rd1^*^Cngb1^-/-^ cell death, when Olaparib does not.
(A) TUNEL-positive cells (magenta) in *wt* and rd1^*^Cngb1^-/-^ retinal cultures. *rd1* retina was left either untreated (Untr.) or treated with INO1001 or Olaparib. Scale bar: 50 μm. (B) Scatter plot showing percent displaying TUNEL-positive cells. Untr. *wt*: *n* = 8; Untr. rd1^*^Cngb1^-/-^: 18; INO1001 rd1^*^Cngb1^-/-^: 14; Olaparib rd1^*^Cngb1^-/-^: 14. Statistical testing: One-way analysis of variance with Tukey’s multiple comparison *post hoc* test performed between *rd1* explant cultures. Error bars represent standard deviation; ^****^P < 0.0001. GCL: Ganglion cell layer; INL: inner nuclear layer; ns: not significant; ONL: outer nuclear layer; PARP-2: poly(ADP-ribose) polymerase-2; rd1^*^Cngb1^-/-^: retinal degeneration 1 cross breed Cngb1 knock-out; TUNEL: terminal deoxynucleotidyl transferase dUTP nick end labelling; *wt*: wild-type.

***Additional Table 1:*** *Information of compounds used in rd1 explant cultures.*

***Additional Table 2:*** *Quantification of calpain-2 activation, calpain activity, PARP activity, and PAR-positive cells in the ONL.*

***Additional Table 3:*** *Quantification of HDAC activity and TUNEL-positive, dying cells in the ONL.*

## Data Availability

*All relevant data are within the paper and its Additional files.*
